# Selective cytotoxicity of a novel immunotoxin based on pulchellin A chain for cells expressing HIV envelope

**DOI:** 10.1038/s41598-017-08037-3

**Published:** 2017-08-08

**Authors:** Mohammad Sadraeian, Francisco E. G. Guimarães, Ana P. U. Araújo, David K. Worthylake, Louis Jr. LeCour, Seth H. Pincus

**Affiliations:** 1 Instituto de Física de São Carlos, Universidade de São Paulo, Caixa Postal 369, São Carlos, SP CEP 13560-970 Brazil; 2 Research Institute for Children, Children’s Hospital, New Orleans, LA 70118 USA; 3 Department of Microbiology, Immunology, and Parasitology, Louisiana State University Health Sciences Center, New Orleans, Louisiana 70112 United States; 4 Department of Biochemistry and Molecular Biology, Louisiana State University Health Sciences Center, New Orleans, Louisiana 70112 United States; 5 Instituto de Física de São Carlos, Universidade de São Paulo, Caixa Postal 369, São Carlos, SP CEP 13560-970 Brazil; 6 Department of Chemistry and Biochemistry, Montana State University, Bozeman, MT 59717 USA

## Abstract

Immunotoxins (ITs), which consist of antibodies conjugated to toxins, have been proposed as a treatment for cancer and chronic infections. To develop and improve the ITs, different toxins such as ricin, have been used, aiming for higher efficacy against target cells. The toxin pulchellin, isolated from the *Abrus pulchellus* plant, has similar structure and function as ricin. Here we have compared two plant toxins, recombinant A chains from ricin (RAC) and pulchellin (PAC) toxins, for their ability to kill HIV Env-expressing cells. In this study, RAC and PAC were produced in *E. coli*, and chromatographically purified, then chemically conjugated to two different anti-HIV monoclonal antibodies (MAbs), anti-gp120 MAb 924 or anti-gp41 MAb 7B2. These conjugates were characterized biochemically and immunologically. Cell internalization was studied by flow cytometry and confocal microscopy. Results showed that PAC can function within an effective IT. The ITs demonstrated specific binding against native antigens on persistently HIV-infected cells and recombinant antigens on Env-transfected cells. PAC cytotoxicity appears somewhat less than RAC, the standard for comparison. This is the first report that PAC may have utility for the design and construction of therapeutic ITs, highlighting the potential role for specific cell targeting.

## Introduction

Pulchellin is a member of type II ribosome-inactivating protein (RIP) family found in the seeds of *Abrus pulchellus tenuiflorus*, with four isoforms (termed P I, P II, P III and P IV)^1, 2^. Other members of this family include abrin, volkesin, ebulin, viscumin and the most extensively studied among all RIPs, ricin^3, 4^. These type II RIPs, with an approximate molecular weight of 56–65 kDa, are composed of an enzymatic A-chain, with the toxic rRNA-specific N-glycosidase activity^5^, covalently linked by a single disulfide bond to a slightly larger B chain^6, 7^, a lectin subunit that mediates cell entry^8^. The A chain of most RIPs, in combination with targeting agents, has anticancer^9^, antiviral^10, 11^ and antifungal activities^12^.

Many studies have been performed with separated toxin A-chain conjugated to carriers capable of delivering them to target cells^9, 13^. This can be achieved by linking them to monoclonal antibodies (MAbs), to form immunotoxins (IT) or other cell-binding conjugates capable of specifically killing cancer cells^14–19^ or some infected cells, such as HIV infected cells^20, 21^.

Ricin (from *Ricinus communis* seeds) was recognized more than a century ago, while *Abrus pulchellus* seeds were discovered within the last 30 years^1, 22^. Our previous studies showed similarities between pulchellin and ricin regarding their structural properties and biological functions^1, 2^, but there have not been any published reports using pulchellin in targeted therapy. To address this, here we study the use pulchellin A chain (PAC) as an anti-HIV IT. Our previous *in vitro* studies have shown that anti-HIV ITs based on ricin A chain (RAC) are highly effective antiviral agents, killing HIV infected T cells with great specificity^23–25^. The envelope glycoprotein (Env) of HIV is the only intact virus protein expressed on the surfaces of virions and infected cells^26^. Therefore, anti-HIV ITs must be targeted to Env^27^. Env consists of gp160 (precursor), gp120 (extracellular domain), and gp41 (transmembrane domain) glycoproteins.

We have conjugated recombinant PAC and RAC to two different anti-HIV monoclonal antibodies (MAbs), anti-gp120 MAb 924^24^ or anti-gp41 MAb 7B2^28^. We performed a side-by-side comparison of their ability to bind, enter and kill HIV infected cells (H9/NL4–3)^27, 29^ or Env-transfected 293 T cells^30^, as well as their non-specific toxicity on uninfected or non-transfected parental cells. The efficacy of anti-gp41 ITs was studied in the presence and absence of soluble CD4 (sCD4)^31^. In this paper we demonstrate that PAC can function within an effective and specific IT, with slightly less efficacy than RAC. An irrelevant antibody conjugated to either RAC or PAC had no effect.

## Results

### Production, characterization and conjugation of toxin A chains to MAbs

**Figure 1:**
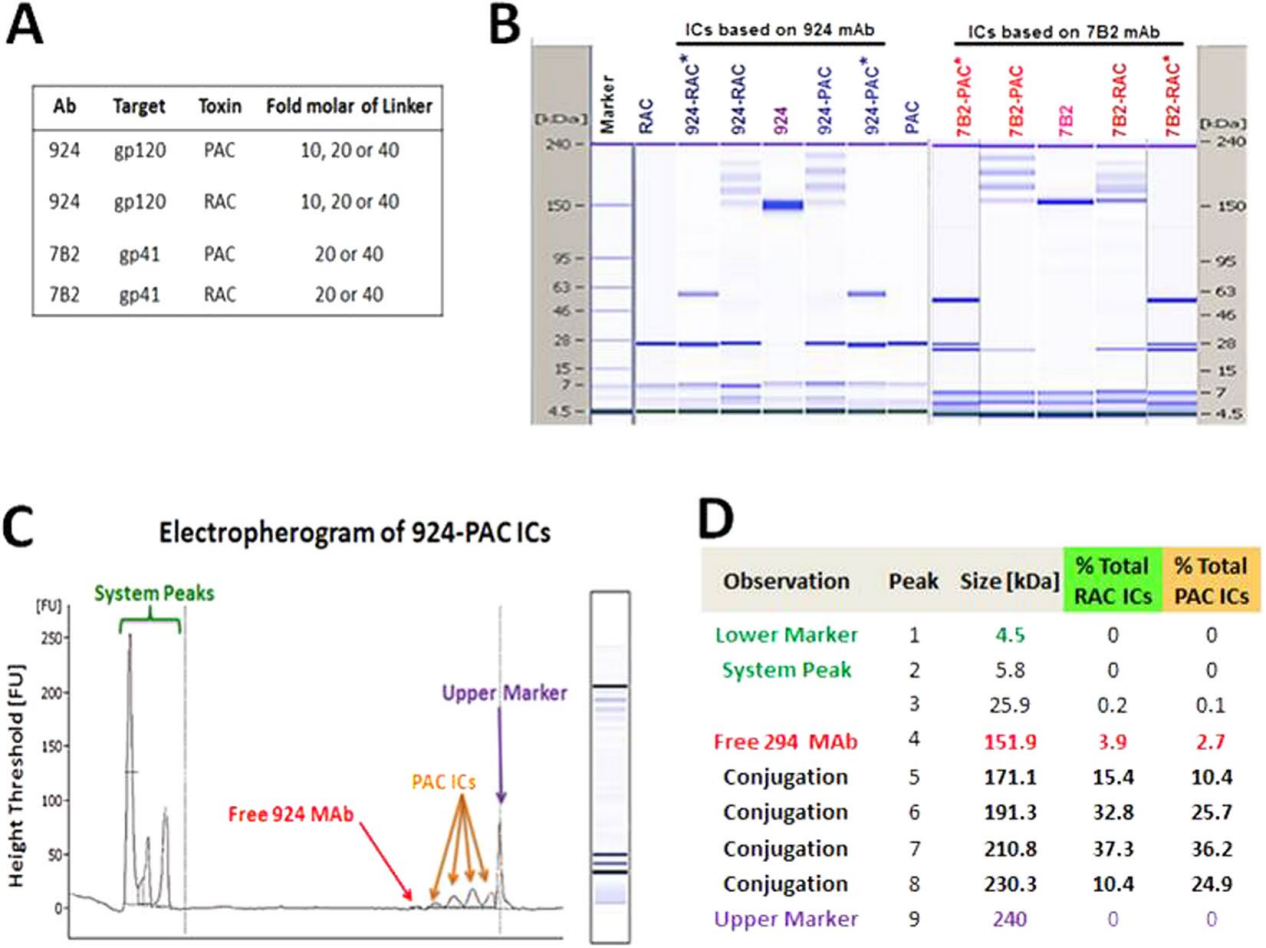
Characterization of immunoconjugates (ICs). (**A**) The table shows components of ICs with different concentrations of LC-SPDP biolinker. The final products include 10 ICs as well as 2 conjugates with irrelevant MAb, as control. (**B**) Microcapillary electrophoresis of reduced and non-reduced ICs. Results are displayed in the familiar format of a coumassie stained gels. Size standards are indicated on the side of each “gel”. PAC, RAC and light chain of 924 MAb represent the same molecular size on the gel. The star symbol (*) means under reducing conditions by using 2-Mercaptoethanol. The ICs represent the conjugation with 40-fold molar excess of LC-SPDP biolinker. In Figure S1, microcapillary electrophoresis of conjugates with different concentration of biolinker are demonstrated. (**C**) Electropherogram of 924-PAC IC, after passing from Ultra-100K centrifugal filter, demonstrates the negligible presence of free MAb as well as MAbs conjugated with 1, 2, 3 or even 4 PAC. (**D**) Peak table of RAC and PAC ICs show the percentage of MAbs conjugated with A chains.

PAC and RAC were produced as recombinant proteins in *E. coli*. After purification of 6xHis tag-removed product by Sephacryl S-200 column, fractions containing purified PAC or RAC were concentrated to 2 mg/ml. Full details are described in Supplementary Information. MAbs were produced in tissue culture supernatants and purified on Protein A agarose beads. MAbs and toxin A chains were tested for purity and size by microcapillary electrophoresis (Fig. 1). Results demonstrate the appropriate size for full length MAbs (H and L chain) with approximately 150 and 157 kDa for 924 and 7B2 MAbs, respectively. Both RAC and PAC are 27 kDa.

We optimized the conjugations of RAC and PAC to MAbs 924 and 7B2, in parallel, by applying different concentrations of LC-SPDP bifunctional cross linker (10, 20 and 40-fold molar excess of LC-SPDP). The results of microcapillary electrophoresis analysis under reducing and non-reducing conditions of the 924-ITs and 7B2-ITs are shown in Fig. 1B. The conjugates with 40-fold molar excess of LC-SPDP biolinker gave major bands of the predicted molecular weights for one and two toxin A chains per antibody molecule (approximate MW, 180,000 and 210,000, respectively) and a minor band of MW 240,000 representing three toxin A chains per antibody molecule (see Supplementary Fig. S1). Under reducing conditions, the conjugates showed three characteristic bands corresponding to light and heavy chains, and the toxin A chains (see Supplementary Fig. S1). By comparing toxin A chains, both RAC and PAC were conjugated equally to 924, however with 7B2 we obtained a superior conjugation with PAC. Then, the conjugates with 40-fold molar excess of biolinker passed from Ultra-100K centrifugal filter. As Fig. 1C shows, by 40-fold molar excess of crosslinker, we could reach to optimum IT with almost eliminated free MAb. In Fig. 1D, the Electropherogram of ITs (40 x ones) shows the total protein mass of RAC and PAC ICs were containing only 3.9% and 2.7% free 924 MAb, respectively.

### Binding of immunotoxins to recombinant antigen

**Figure 2:**
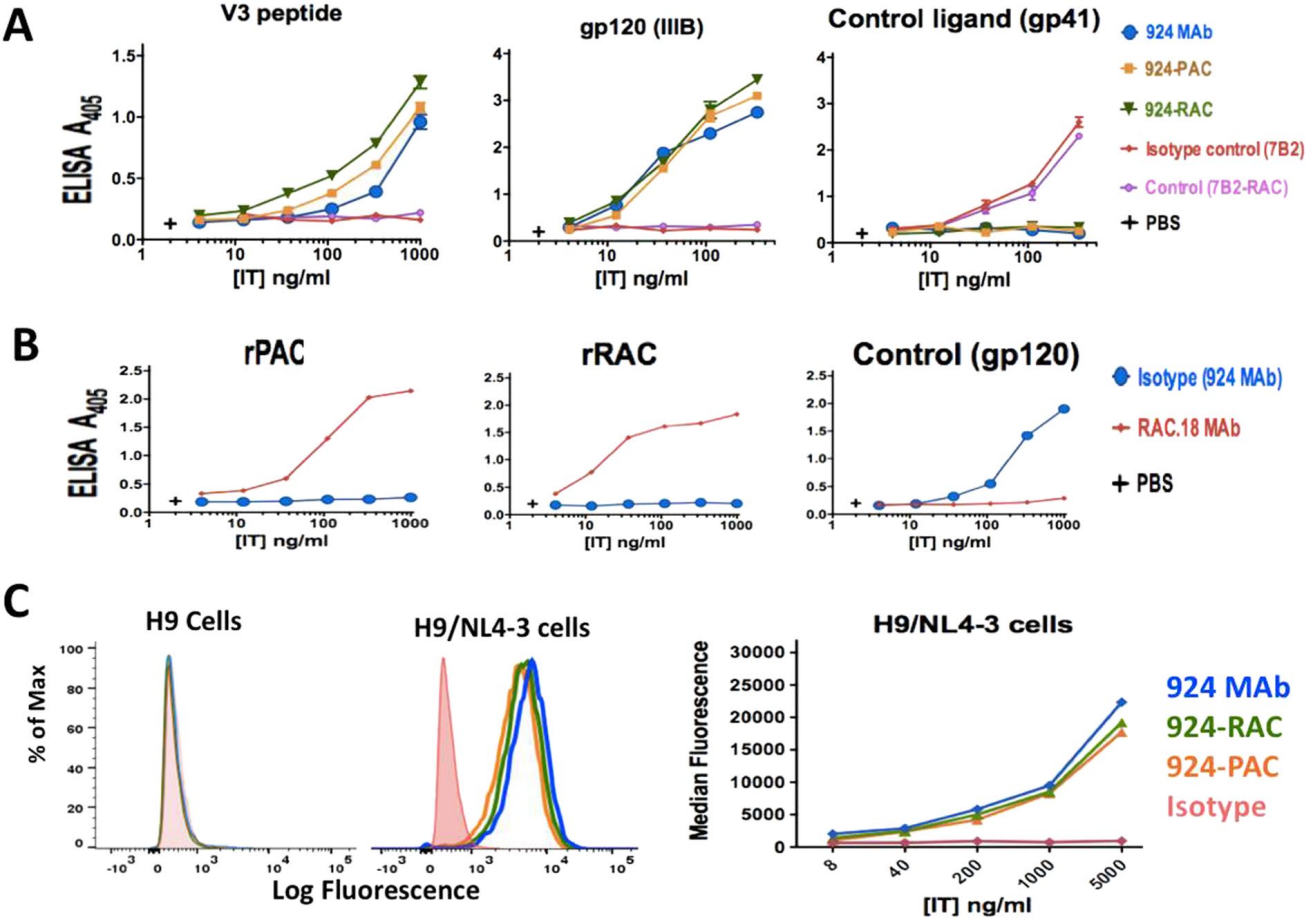
924 based-ITs bind to recombinant antigens and native antigens on the HIV infected cells. (**A**) ELISA plates are coated with a synthetic V3 loop peptide, recombinant gp120 and gp41 peptide as a control ligand. The results show the binding of 924 MAb and 924-ICs to the either gp120 or V3 peptide, but not the unrelated isotype controls. (**B**) ELISA plates are coated with recombinant RAC, PAC and recombinant gp120 as a control ligand. The results demonstrate the binding of anti-ricin A chain MAb (RAC18) to rPAC and rRAC, but not the unrelated isotype control. In panels (**A** and **B**) the Ab binding is detected with AP-conjugated goat anti-mouse IgG. Where no error bars are visible they are obscured by the symbol. Results are representative of means of duplicate values with at least three different assays (varying by Ab, ITs, or Ag tested). (**C**) Flow cytometry histograms for binding of 924 MAb and 924 based-ITs to uninfected H9 cells, as control, and persistently-infected H9/NL4-3 cells. Binding was detected with Alexa488 conjugated goat anti-mouse IgG. On the right, IT binding to H9/NL4-3 cells is plotted as median fluorescence versus IT concentration. The results are representative of three independent experiments. Isotype control is shown as red shaded histogram.

**Figure 3:**
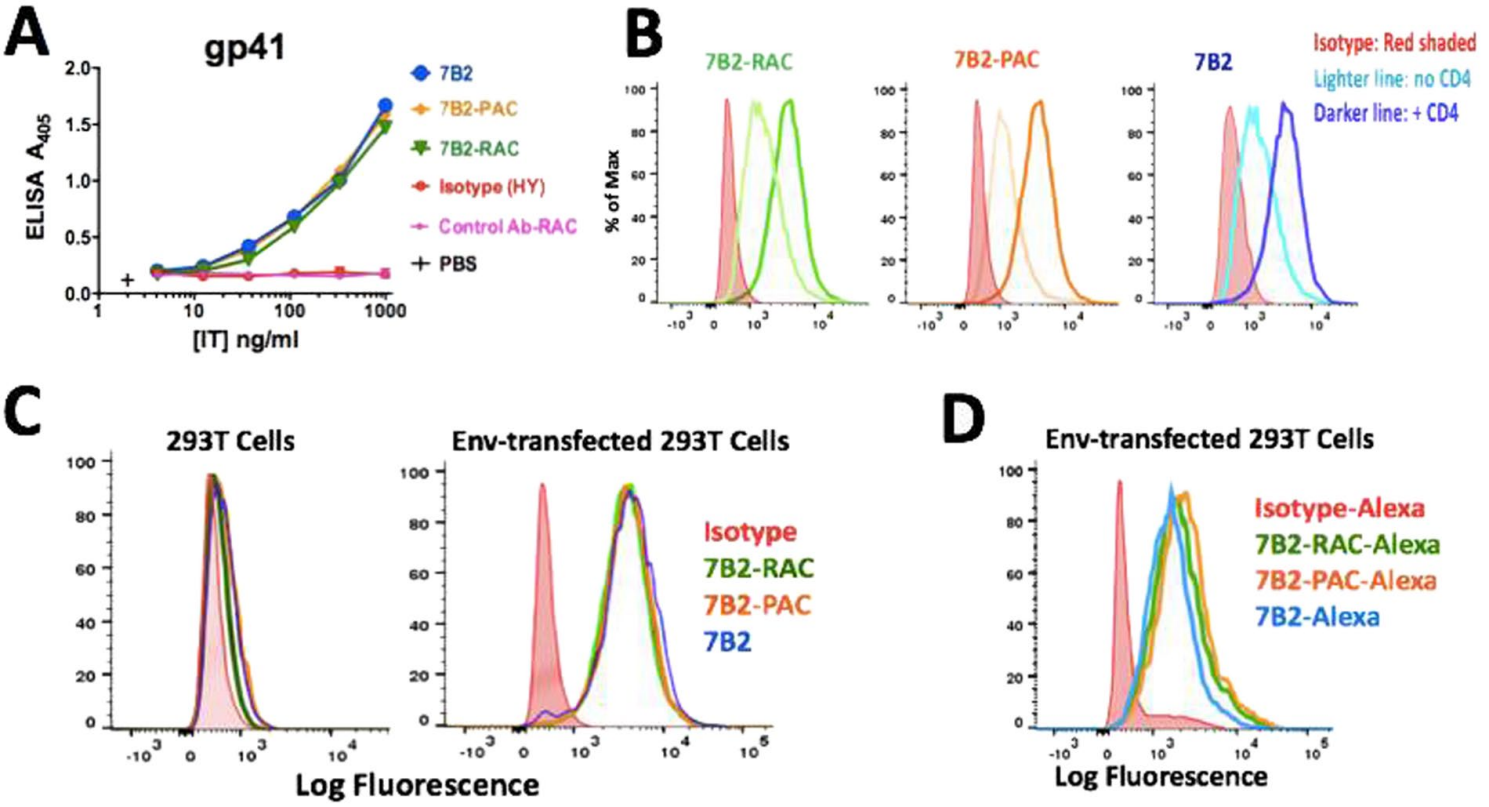
Binding ability of 7B2 based-ITs to gp41 antigen by using ELISA and flow cytometry. (**A**) ELISA plates coated with gp41 Ag, as a peptide representing 7B2′s epitope. Binding of the 7B2 based-ITs is detected by AP-conjugated goat anti-human IgG. Results are representative of means of triplicate values with three individual experiments. (**B**) Using FITC-secondary immunofluorescence, we detect binding of ITs to Env-transfected 293 T cells in the presence (**darker line**) or absence (**lighter line**) of sCD4 (CD4-183). Isotype control is shown as red shaded histogram. (**C**) IT binding to 293 T cells and transfected 293 T cells with 92UG037.8 gp160 (293 T/92UG) was detected by using FITC-secondary immunofluorescence. Results are representative of at least three independent experiments. (**D**) The flow cytometry histogram of Alexa488-labeled ITs by using Env-transfected 293 T cells incubated in PBS + 1% BSA + sodium azide (0.2%). Results are representative of three individual experiments. Isotype control (chimeric RAC18-alexa488) is shown as red shaded histogram.

By using an indirect ELISA, we tested the ability of purified MAb 924 and 924-based ITs to bind recombinant gp120(IIIB) antigen and V3 loop peptide. In parallel, the binding ability of MAb 7B2 and 7B2-based ITs were examined by using the gp41 loop peptide antigen. In Figs 2A and 3A, all the purified MAbs and conjugates bound to the appropriate antigens, while the isotype controls (MAbs and MAb-RAC) and negative control (plate coated with unrelated control ligand) did not, demonstrating that immunologic specificity of the antibodies in the ITs was unaffected by the conjugation process. Moreover, the results demonstrate that the different concentrations of biolinker did not significantly affect the function of the conjugated MAb (see Supplementary Fig. S1). Besides, we examined the ability of anti-ricin A chain MAb RAC18 to bind recombinant RAC (rRAC) and recombinant PAC (rPAC). As has previously been reported^32^, RAC MAb18 bound to rRAC, with no binding to the isotype control MAb 924. Interestingly, RAC MAb18 showed a binding ability to rPAC as much as rRAC (Fig. 2B).

### ITs bind to Env expressed on the surface of infected and transfected cells

We examined binding to native Env by flow cytometry, using FITC conjugated goat anti-human or Alexa488 conjugated goat anti-mouse IgG secondary antibody to detect IT binding. 924-based ITs bound to persistently infected H9/NL4–3 cells (Fig. 2C). 7B2-based ITs bound to H9/NL4-3 cells^28^ as well as to 293 T cells transfected with 92UG037.8 gp160 (Fig. 3B–D). ITs did not bind uninfected H9 or 293 T cells, confirming the specific cell binding of ITs. Addition of sCD4-183 enhanced binding of 7B2-based ITs to Env-transfected 293 T cells (Fig. 3B), as has previously been reported for RAC-7B2^25, 31^. We directly labeled ITs to Alexa488 for later experiments. Figure 3D shows direct fluorescence with the ITs and naked 7B2 after Alexa488 labeling.

### Cell binding and internalization of immunotoxins

**Figure 4:**
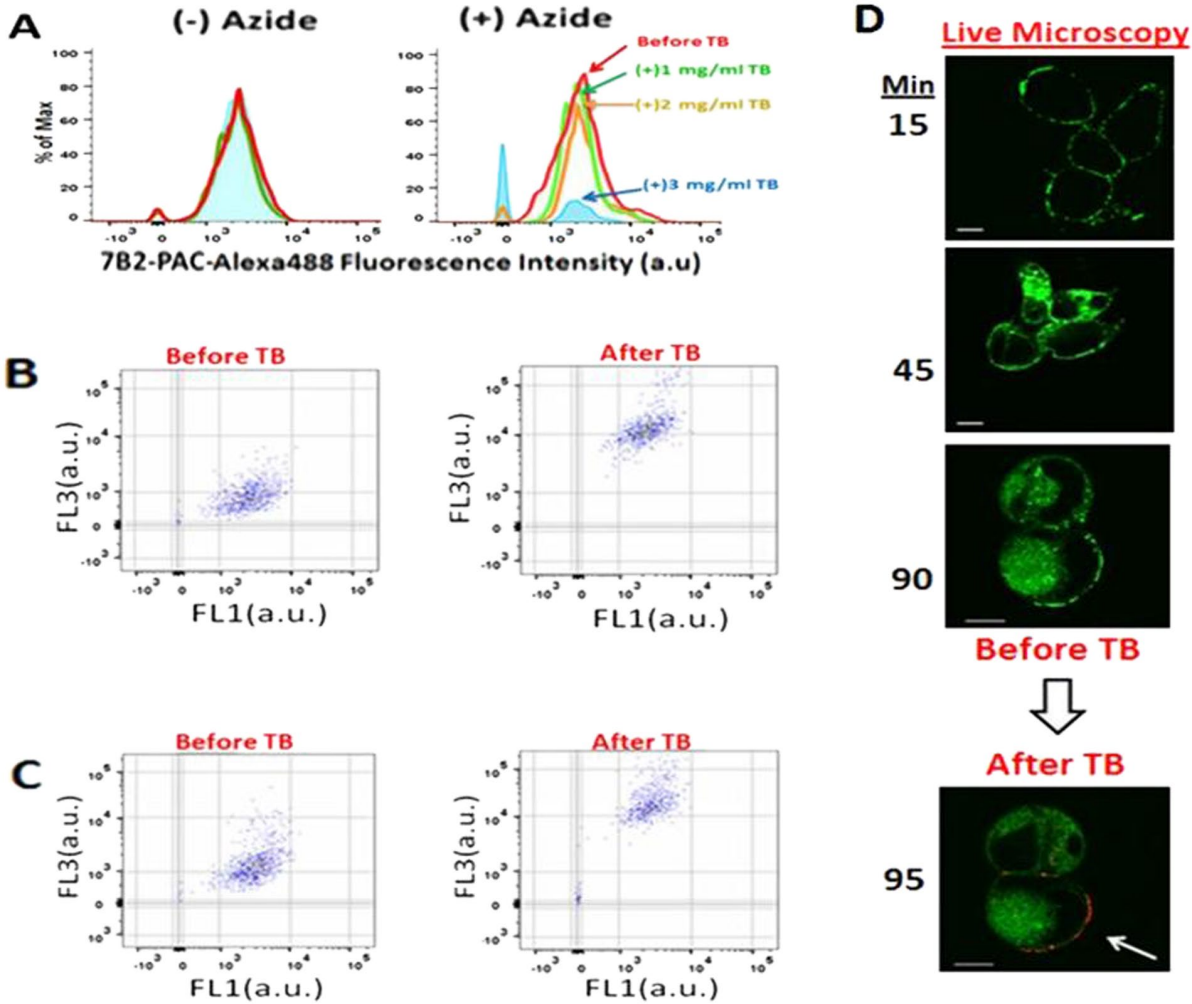
Distinguishing the internalized ITs from those bound on the cell surface by using quenching effect of Trypan Blue (TB). All the flow cytometry and microscopic experiments are in the presence of sCD4-183 (300 ng/ml) (**A**) Before incubation with ITs, the viability of cells was 96%. The diagrams show percentage of 7B2-PAC-Alexa488 that remained fluorescent after the addition an increasing concentration of TB (1, 2 and 3 mg/ml). In the presence of NaAz, 7B2-PAC can only attach to the cell membrane, without cell internalization. As the right diagram demonstrates, the addition of an increasing concentration of TB shows that a concentration of 3 mg/ml of TB is sufficient to quench the extracellular fluorescence. In the absence of NaAz, due to the IT internalization, the fluorescence intensity of ITs remain intact from quenching by TB (**B,C**). Dot plots of cells incubated with 7B2-PAC-Alexa488 (**B**) or 7B2-RAC-Alexa488 (**C**) in the absence of NaAz, analyzed by flow cytometry before TB addition and 2 hr after that. Before TB addition, the dot plots of the cell population incubated with Alexa-ITs, either adherent to plasma membrane or internalized, are emitting green fluorescence (FL1). TB cannot enter the live cells, therefore, after TB addition the green fluorescence emission (FL1) is not quenched, while we observe the upshift of cell population corresponding to the cells with damaged membrane (ie. 4% dead cells) and Alexa-ITs adherent to the membrane which emitted red fluorescence (FL3) after quenching. Results are representative of two independent experiments. (**D**) Live confocal microscopy by taking images from a series of different regions started with time after the addition of Abs, generally 50 observations during the 90 min period. Live cells incubated with 7B2-PAC-Alexa488 demonstrate the presence of IT on the cell surface after 15 min. Following 90 min, 7B2-PAC is observable both internalized and on the cell surface. By imaging the same region, after 5 min TB addition, only the green fluorescence on the cell surface is quenched and emitting red fluorescence (shown by white arrow). The green and red fluorescences are detected by band-pass filters 530 ± 30 nm and 650 ± 13 nm, respectively.

To quantify the internalization of ITs by flow cytometry, the Env-transfected 293 T cells were incubated with Alexa-labeled ITs in the presence or absence of 0.05% sodium azide (Na Az). Prior to cytometry external fluorescence was quenched with trypan blue (TB). Given that TB enters dead cells and may interfere with the fluorescent analysis, we confirmed high cell viability (96%). In the presence of NaAz, ITs cannot be internalized, they only bind to antigens on the cell surface. The addition of an increasing concentration of TB showed that 3 mg/ml of TB was sufficient to quench extracellular fluorescence (Fig. 4A). However in the absence of NaAz this concentration of TB caused no quenching (Fig. 4A), indicating the Alexa-labeled ITs were internalized, although we cannot rule out a small amount was bound to the cell surface. Figure 4B demonstrates the population of cells in the absence of TB, with 7B2-PAC-Alexa488, either bound to the cell membrane or internalized. We also observed a negligible population of cells without Alexa-labeled ITs. After addition of TB, the green fluorescence emission was not quenched, signifying the 7B2-PAC-Alexa was internalized. Moreover, the upshift of cell population corresponded to the cells with damaged membrane (dead cells) and Alexa-labeled ITs adherent to the membrane which emitted red fluorescence after quenching. Figure 4C shows the same results for 7B2-RAC-Alexa488.

### Live imaging of ITs during binding and internalization

Internalization of ITs was confirmed by live confocal microscopy of Env-transfected cells with 7B2-PAC-Alexa488. After the addition of IT, live imaging included approximately 50 observations during the 90 min period. In Fig. 4D, after 15 min of incubation, the IT was observable on the cell surface, and after 30 min, was identified intracellularly as small vesicles. Following 90 min, the imaging of one specific region before and after TB addition demonstrated quenching of the green fluorescence on the cell surface and emitting red fluorescence (shown by white arrow in Fig. 4D).

### Confocal microscopy of binding, internalization and intracellular localization of ITs

**Figure 5:**
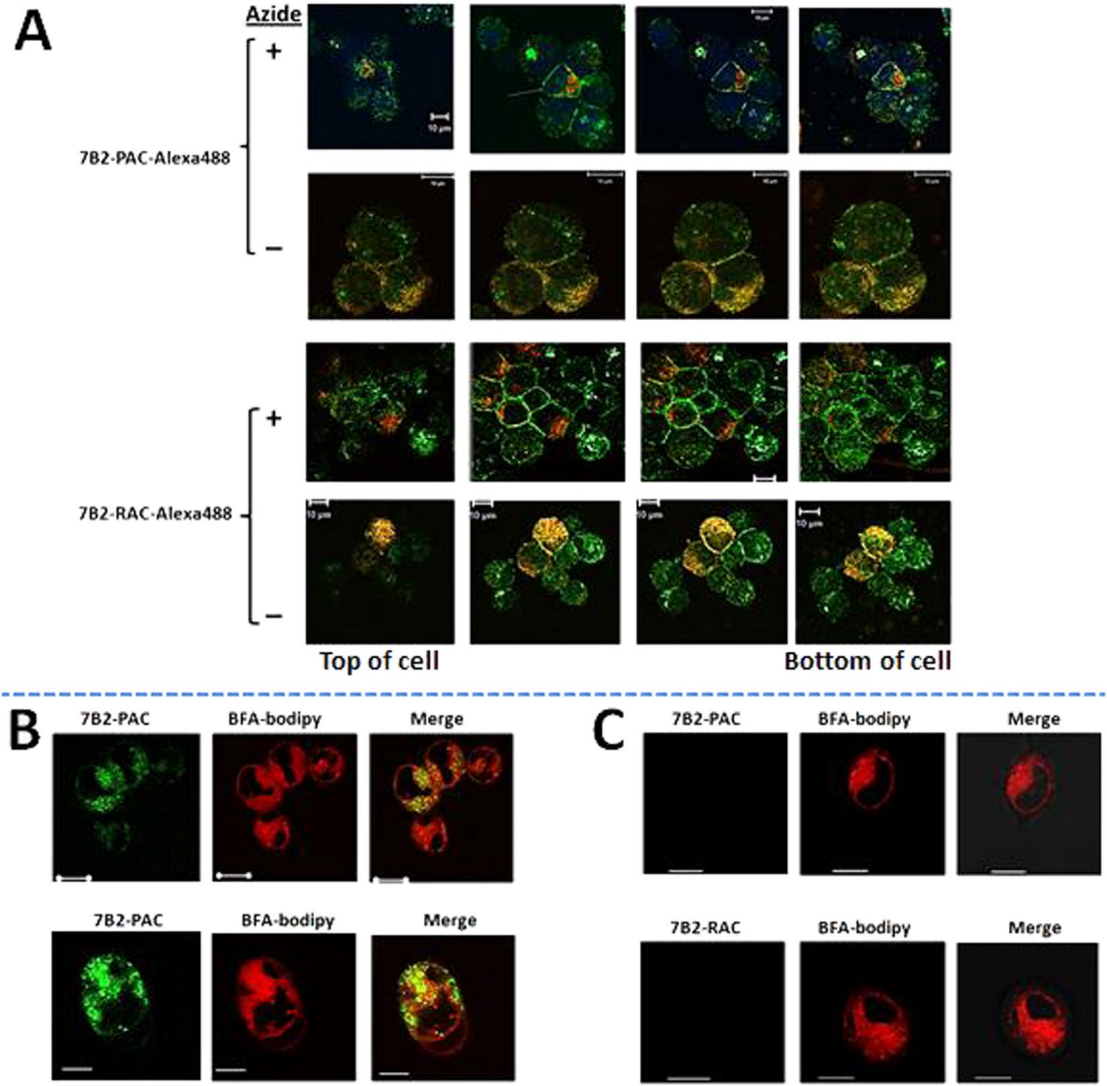
Binding, internalization and intracellular localization of fluorescent ITs. (**A**) Env-transfected 293 T cells were incubated with cold PBS in the presence or absence of 0.02% sodium azide. 45 min later, Alexa488-ITs were separately added to each set of slides in the presence of sCD4-IgG2. The slides were counterstained with BFA-bodipy, incubated an additional 60 min under the same conditions. Five min later, cells were washed 3X with cold PBS in the presence or absence of azide. The cells were fixed in 2% paraformaldehyde or one drop SlowFade Gold Mountant DAPI. Cells were observed with a 62X oil-immersion objective. Z-stack images collected at 1 µm sections. The bottom of cells represents the closest plane to the slide. ITs are green, ER and Golgi are red. Some samples have nuclei stained with Hoechst Dye (Blue). Colocalization of red and green dyes appears yellow. The white bar indicates 10 µm. (**B,C**) A comparison between Env-transfected 293 T cells (**B**) and 293 T cells (**C**) incubated with the same description in panel (**A**) but in the absence of sodium azide, in order to show the specific targeting the transfected cells. BFA-bodipy was used to demonstrate accumulation of ITs on the ER and Golgi.

The localizations of 7B2-PAC and 7B2-RAC were studied by non-quantitative confocal microscopy. Env-transfected 293 T cells were incubated with Alexa488-ITs and BFA-bodipy under physiologic conditions, or in the cold with sodium azide to block the entry of IT into the cell. Serial vertical sections are shown in Fig. 5. In the presence of azide (Fig. 5A), ITs only bound to the cell surface, whereas under physiologic conditions 7B2-PAC or 7B2-RAC were observed on the cell surface and internalized. The areas of yellow signal demonstrate the colocalization of internalized IT (green signal) on the ER and Golgi colored by BFA-bodipy (red signal).

### Immunotoxins based on PAC effectively and specifically kill infected/transfected cells

**Figure 6:**
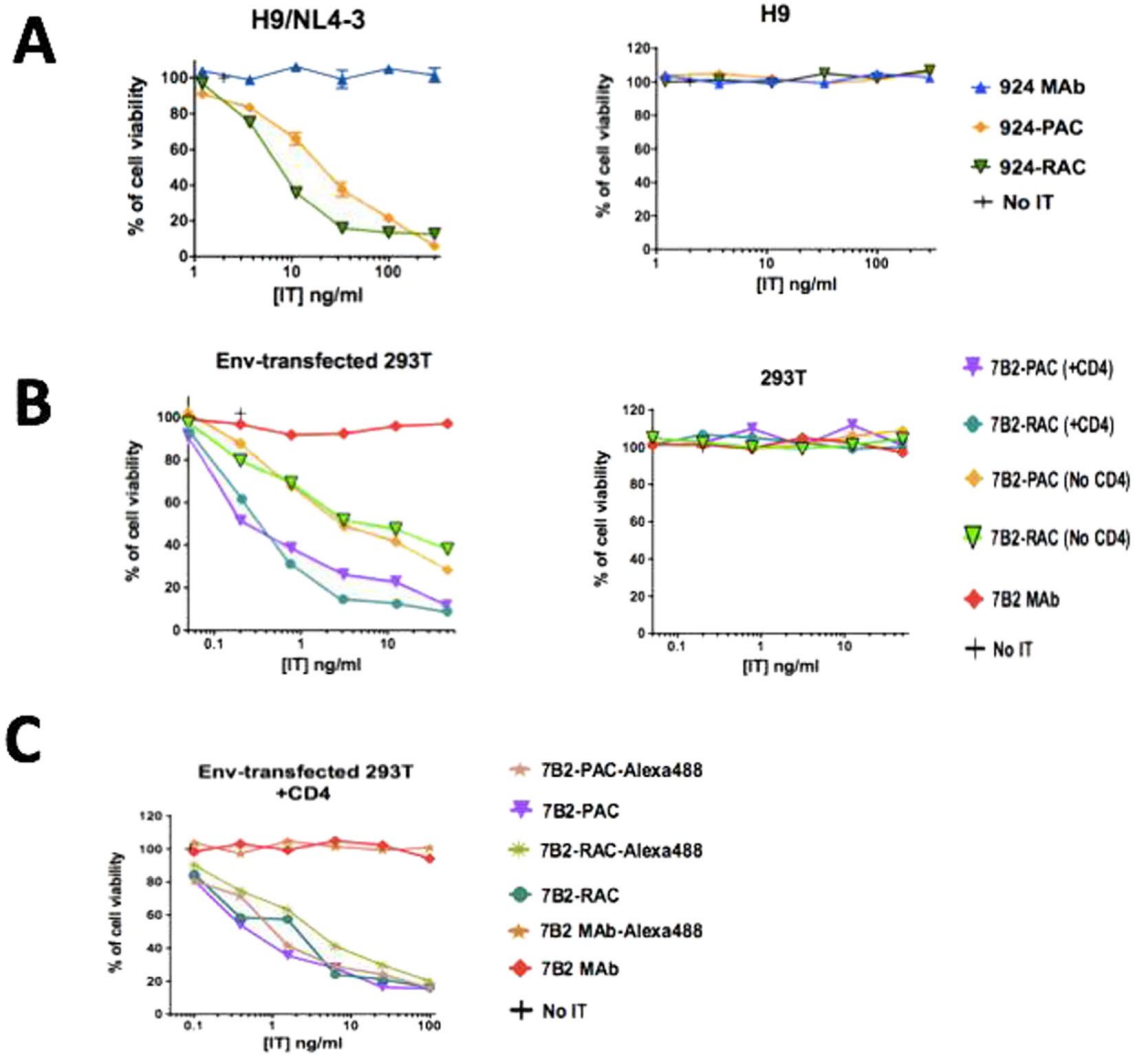
Comparing the cytotoxicity and targeting of ITs by direct cytotoxicity assay. (**A**) The cytotoxicity of 924-based ITs was assayed by using persistently-infected H9/NL4-3 cells. 924-RAC appears more cytotoxic than 924-PAC. ITs did not have cytotoxicity on T-cell lymphoma (H9) cells, signifying ITs have specific targeting. (**B**) The cytotoxicity of 7B2-based ITs to Env-transfected 293 T cells (293 T/92UG) in the presence or absence of sCD4 (anti-gp120) was assayed. The results show that 7B2-RAC and 7B2-PAC had equivalent cytotoxicity. The presence of soluble CD4 (300 ng/ml) had a significant enhance on the cytotoxicity of ITs. (**C**) The cytotoxicity of ITs was compared before and after labeling in the presence of sCD4 (300 ng/ml). Results show mean and standard error of triplicate samples. Results are representative of three individual experiments. Where no error bars are visible they are obscured by the symbol.

To test biological activity of ITs, we used a direct cytotoxicity assay in two experiments: In the first experiment, we used H9/NL4-3 infected T cells to test the cytotoxicity of 924-based ITs. In the second, we assayed the cytotoxicity of 7B2-based ITs, including Alexa-labeled and unlabeled versions, to Env-transfected 293 T cells in the presence or absence of sCD4. In each experiment, the cells were incubated with differing concentrations of ITs or MAbs as control. The other controls included; no cells (background) and cells in the absence of IT. Cell viability was measured after 3 days by MTS dye reduction, with a decrease in A_490_ indicating cell death (Fig. 6 and S2). The results demonstrate the efficacy and specificity of each IT for the infected cells, but not uninfected cells. Although 924-PAC appeared less efficacious than 924-RAC, these results were not observed with 7B2-PAC and 7B2-RAC. The amount of LC-SPDP used for the conjugation had little effect on IT efficacy (Fig. S2). The cytotoxicity of ITs used in imaging analyses was unaffected by Alexa conjugation (Fig. 6C).

## Discussion

To develop improved ITs, different toxic domains have been used, aiming for higher toxic efficacy or fewer undesired effects^14^. Pulchellin toxin is a type II RIP, structurally similar to ricin^22, 33^. Moreover, the A chains of both ricin and pulchellin contain only one cysteine residue for forming an interchain disulfide bridge^2, 34^. In spite of the similarities in the structure of ricin and pulchellin, the anti-viral and anti-tumor activities of RAC have been studied well^3, 35, 36^, while there are no published data to exploit the feasibility of using PAC in targeted therapy. Here we have explored the function of PAC as an IT candidate, when coupled to MAbs to the HIV envelope, the only intact virus protein expressed on the surfaces of virions and infected cells.

We have conjugated two different anti-Env Abs to RAC and PAC, then tested their cytotoxic activity on either persistently infected or transfected cell lines. The results clearly indicate the efficacy of PAC-containing ICs. The comparative efficacy of PAC vs the well-studied toxic moiety RAC is not fully resolved, with one set of experiments using 924 MAb showing PAC to be slightly less efficacious than RAC, but the set of experiments with 7B2 ITs showing no difference. Within the range we tested, the concentration of bifunctional crosslinker did not appear to have a significant effect on the binding ability of the conjugates (see Supplementary figures S1 and S2). There is only one free cysteine on PAC (Cys247) which reacts with MAb-linker rapidly in high yield without diminishing biological activity of toxin. SPDP can crosslink with multiple alternative amino groups on the MAb^37, 38^. This may affect on the MAb binding ability (not the A-chain toxicity), while we have shown by ELISA and FACS which is not affected.

In this study we tested the 924 ITs on persistently infected H9/NL4-3 cells. The Env expressed by this virus is the prototypical laboratory strain IIIB, and MAb 924 binds uniquely to the tip of the V3-loop of this isolate. MAb 7B2 binds a well conserved epitope in the helix-loop-helix region of gp41. The peak table in Fig. 1D, demonstrates a stoichiometric similarity between PAC and RAC ICs, regarding in the number and percentage of the linked A-chains. This similarity of stoichiometric heterogeneity may not affect the interpretation of the cytotoxicity assay during comparison between RAC and PAC ICs. The efficacy of 7B2-RAC ITs has been studied extensively in a variety of HIV infected cells, and more recently in Env-transfected 293 T cells. The efficacy of 7B2 ITs has been demonstrated *in vivo* ^28, 39^. Here we have used cells stably transfected to express fully functional envelope (gp160, gp120 and gp41) from HIV clade A clinical isolate gp160 (293 T/92UG)^30^ and shown that 7B2-RAC and 7B2-PAC are equivalently cytotoxic. As we have observed previously^31^, soluble CD4 enhances epitope exposure and IT efficacy. By comparing the susceptibility of these two cell lines, as we have shown before^27^, 293 T/92UG cells are significantly more sensitive to the IT than H9/NL4-3 cells. These differences in IT-mediated killing, between transfected 293 T/92UG cells and infected H9/NL4-3 cell lines, are unrelated to the binding of the MAb to the target cells. As we have explained previously^27^, discrepancies among these two cell lines may reflect differences resulting from transfection versus infection and/or laboratory-adapted versus clinical isolates.

In this study, we demonstrate that PAC stands outs as feasible candidate for the design and construction of effectively specific ITs. In this case, the immunogenicity of PAC will be a matter of great interest; whereas the development of anti-pulchellin Abs will be a major limitation for pulchellin-based immunotoxins. RAC MAb 18 has a common motif of QXXWXXA on the structure of RAC^40^ and PAC which fold into part of the enzyme active site^2^. We have demonstrated that RAC MAb 18 binds to not only rRAC also rPAC (Fig. 2B). This finding helps us for our future study on the immunogenicity of PAC.

Our previous microscopy studies have revealed the intracellular localization and trafficking of whole pulchellin and ricin toxins^32, 41^. Herein, we have used flow cytometry and confocal microscopy to study cell binding, uptake, and intracellular localization of 7B2-PAC. We have used Env-transfected 293 T cells for microscopic study, because these cells are adherent in compare to suspension H9 lymphoma cells^42^. We have shown that 7B2-PAC binds to the cell surface and is internalized, possibly localizing to ER and Golgi, within one hour. We are now studying the intracellular trafficking of PAC immunotoxins.

This is the first demonstration that PAC may have utility for the design and construction of therapeutic ITs, highlighting the potential role for specific cell targeting not only for AIDS also cancer treatment.

## Materials and Methods

### Cells and reagents

H9/NL4-3 cells are laboratory-adapted and persistently infected with the NL4-3 molecular clone of HIV^27, 29^ and maintain a productive infection in all cells during passage in tissue culture. Uninfected H9 cells, a human CD4 + lymphoma cell line, were obtained from Dr. M. Reitz (Institute of Human Virology, Baltimore, MD). H9 and H9/NL4-3 cells were maintained at 37° in 5% CO_2_ in RPMI 1640 medium with 10% fetal bovine serum (Gibco Invitrogen, Grand Island NY) as described elsewhere^23^.

293 T cell lines stably express clade A clinical isolate 92UG037.8 gp160 as native gp120/gp41trimers (293 T/92UG)^30^. 293 T cells were used as uninfected control cells. The transfected and non-transfected 293 T cells were maintained at 37° in 5% CO_2_ in DMEM medium with 10% fetal bovine serum (Gibco Invitrogen, Grand Island NY). In this paper, Env-transfected cells refer to the 293 T cells stably transfected with 92UG037.8 gp160.

### Antibodies

MAb 924 was produced from a mouse immunized with a vaccinia virus recombinant containing a gene encoding the HIV gp160, as described elsewhere^24^. This IgG1 antibody reacts with the V3 loop of HIV gp120 of LAV/HTLV-IIIB isolate. MAb 7B2 (Genbank accession numbers JX188438 and JX188439) is a human IgG1 which binds HIV gp41 at AA 598–604 (CSGKLIC) in the helix-loop-helix region^28, 31^. HY (Genbank accession numbers JX188440 and JX188441), an affinity matured version of the anti-CD4 binding site Ab b12^43^. Murine and chimeric version of anti-ricin A chain MAb RAC18 and Alexa 488-conjugated RAC18 have been described elsewhere^40^, as has the isotype control 924 MAb^32^. MAbs were purified from hybridoma supernatant on Protein A agarose beads (Invitrogen) and eluted with 0.1 M glycine, pH 2.5, immediately neutralized, and dialyzed vs PBS. Two types of soluble CD4 were used to observe CD4-mediated effects^31^; CD4-183 contains the first two domains of CD4 including the region in domain 1 that binds the HIV coat protein gp120. CD4-IgG2 is a tetrameric fusion protein comprising human IgG2 in which the Fv portions of both heavy and light chains have been replaced by the V1 and V2 domains of human CD4. Goat anti-human IgG (Invitrogen) was conjugated to either alkaline phosphatase (AP) or fluorescein isothiocyanate (FITC). Goat anti-mouse IgG (Biacore) (heavy + light chains) secondary antibody was conjugated to Alexa-488.

### Cloning, expression and purification of recombinant toxin A chains

Recombinant pulchellin A-chain (rPAC) was generated by PCR using the pulchellin cDNA isoform PII^2^, accession number EU008736.1, as template. In our previous study, the rPAC had a 6xHis-tag at its N-terminal end^2, 34^. In this experiment, we designed a new construct containing a tobacco etch virus protease (TEV) cleavage site for proteolytic removal of the 6xHis-tag after purification. Specific primer sequences were the following: rPAC-forward (5′ CCCC**ATG**GCTAGCGAGGACCGGCC) and rPAC-reverse (5′GTGCTCGAGTTAATTTGGCGGATTGC), including the restriction sites for *Nco*I and *Xho*I, respectively. PCR products were cloned into the pGEM-T (Promega) vector and its sequence was confirmed using an ABI Prism 377 automated DNA sequencer (Genescript). Recombinant RAC was produced in BL21(DE3)RIL cells (Novagen) from plasmid pETtrx, which encodes *E. coli* thioredoxin and a TEV protease-cleavable 6xHis affinity tag in frame with and N-terminal to the RAC coding sequence. The target sequences of RAC and PAC were subcloned into pET28a(+) vector (Novagen).

Expression and purification of PAC and RAC is described in supplementary data. Briefly, the recombinant PAC and RAC were produced in *E. coli* Rosetta (DE3), and purified by HisTrap Nickel column. The His-tag was cleaved with TEV protease, and the tag-less toxin A chain was purified on a HiPrep 26/60 Sephacryl S-200 column.

### Conjugation of Abs to RAC and PAC

HIV MAbs 924 and 7B2 were conjugated separately to PAC and RAC by using a modification of the protocol described elsewhere^23, 24^. Optimization of the concentration of heterobifunctional cross-linking reagent succinimidyl 6-[3(2-pyridyldithio) propionamido] hexanoate (SPDP, Pierce) was carried out for conjugation between amino groups (on lysine and at the N-terminus) on antibody and the single free cysteine on A-chain toxin^37, 38^ by applying three different concentrations of LC-SPDP biolinker (10, 20, and 40X molar excess relative to MAb), as described in supplementary data, figure S1. After 2 hr of incubation at room temperature, the MAbs and SPDP were separated on a Zeba desalting column (Pierce) equilibrated with PBS.

PAC and RAC (1 mg in 0.5 ml), which were stored at –80 °C in reduced form, were desalted on Zeba column. The RAC/PAC and MAb-SPDP were mixed separately, concentrated to 0.5 ml and incubated overnight at 4 °C. Individual fractions were analyzed by microcapillary electrophoresis (Agilent Bioanalyzer, GE Healthcare). After the conjugation reaction, the removal of unreacted A-chain toxin and holotoxins were achieved by using an Amicon Ultra-100K centrifugal filter (Millipore). The concentrations were measured by bicinchoninic acid protein assay (Pierce, Rockford, IL) and confirmed using OD280 reading by Nanovue UV Spectrophotometer (GE Healthcare, Piscataway, NJ), before and after passing from filter.

### ELISA

ELISAs were performed for Ag-binding specificity analysis and titration of purified MAbs and ITs in wells coated with antigen (1 μg/ml), as described elsewhere^25^. The gp41 antigen was a linear peptide HIV-1 consensus clade B sequence [LGIWGCSGKLICTT] representing the epitope of 7B2. Gp120 antigen was a recombinant protein expressed in mammalian cells. Recombinant gp120 antigen represented HIV isolate IIIB (gift from Genentech, S. San Francisco, CA). The synthetic V3 loop peptide represented the V3 sequence of strain IIIB (amino acids AA 297–330; numbering according to reference 44, TRPNNNTRKSIRIQRGPGRAFVTIGKIGNMRQAH. Binding of antibody to the antigen was detected with AP-conjugated secondary antibodies: goat anti-mouse IgG (H + L chain specific) for HIV MAb 924 as well as 924 based-ITs; or goat anti-human IgG (H + L chain specific) for HIV MAb 7B2 as well as 7B2 based-ITs (all from Zymed Laboratories, South San Francisco, CA). Data are reported as optical density at 405 nm and represent means of triplicate values with three independent experiments.

### Immunofluorescence assay

We used indirect immunofluorescence and flow cytometry to analyze binding of immunotoxins to either persistently infected H9/NL4-3 cells, or Env-transfected 293 T cells. 8 × 10^4^ cells were added to IT to reach the final concentrations of 5 ng/ml to 5 µg/ml. Cells were incubated 1 hr at room temperature. Cells were washed, and then stained with Alexa488-conjugated goat anti-mouse IgG secondary antibody (2 μg/ml) for 924 based-ITs; or FITC conjugated goat anti-human IgG secondary antibody (2 μg/ml) for 7B2 based-ITs for 1–4 hr, washed twice and fixed in 100 μl of 2% paraformaldehyde. After a minimum of 4 hr, 150 μl of PBS was added. Cells (10,000) were studied on a Becton-Dickson LSR II with HTS plate reader, analyzed by Flow-Jo software (Treestar Inc). Forward scatter (FSC) and side scatter (SSC) gated data are represented as either overlayed histograms or as graphs of mean fluorescence. The conjugates or MAbs did not bind to uninfected H9 cells nor untransfected 293 T cells.

### Alexa Fluor 488 labeling of ITs

Labeling was carried out by mixing 1 ml of each IT at ~1 mg/ml, in sodium phosphate buffer 50 mM, pH 7.5, NaCl 0.1 M, with 100 µg Alexa Fluor 488 as N-hydroxysuccinimide (NHS) salts (Invitrogen Molecular Probes, Eugene, OR). The reaction was maintained for one hour in the dark at room temperature with continuous stirring. Labeled protein was separated from unconjugated dye on Zeba 7KD cutoff desalting columns (Pierce, Rockford, IL). The degree of labeling was determined by measuring the A280 and A495 of the conjugates. ITs were characterized before and after labeling, with aforesaid protocols.

### Measurement of internalization by flow cytometry

Env-transfected 293 T cells (1.5 × 10^5^) were placed in 100 ml in round-bottom 96 well plates (Costar, Lowell, MA) in the presence or absence of 0.05% NaAz for studying, respectively, the binding or internalization of ITs by flow cytometry. Cells were stained with 1.0 μg/ml Alexa488-labeled ITs in the presence of 1.0 μg/ml sCD4-183 in serum-free medium for 4 hr at 4 °C. As negative controls, two wells either were stained by ChRAC18 or were left unstained. Cells were washed twice then 250 µl of PBS was added, in the presence or absence of 0.05% NaAz. The percentage of cell viability was analyzed by optical microscopy by incubating with 0.4% Trypan Blue (TB, Sigma) solution diluted in PBS to perform the TB exclusion test with counting in a Neubauer chamber. In order to confirm internalization of ITs to the cells, we used a modification of the protocol described elsewhere^45–47^. An increasing concentration of Trypan Blue from 1 to 3 mg/ml was used to quench the extracellular fluorescence. Cells were analyzed under a Becton-Dickson LSR II (BD, Franklin Lakes, NJ) both prior and 2 min after TB addition. 10000 events were collected and data analyzed by Flow-Jo software (Treestar, Ashland, OR). Green fluorescence (FL1) of internalized Alexa488-labeled ITs was collected using a 530 ± 30 band-pass filter; red fluorescence emitted from TB bound to Alexa488-labeled ITs on the cell surface (FL3) was collected by using a 650 ± 13 band-pass filter. The data were collected using linear amplification for FSC and SSC, and logarithmic amplification for FL1 and FL3.

### Live confocal imaging

Live cell imaging was performed as described elsewhere^32^. All the images, including those used for quantitative analyses, were obtained with an inverted Zeiss LSM 510 microscope, a 63 × 1.4 NA oil-immersion objective, and Zeiss LSM software. Heated (37 °C) stages and objectives were used on microscope. One day prior to imaging, 10^4^ Env-transfected 293 T cells were seeded into 35 mm culture dishes with 0.17 mm thickness glass bottom (MatTek, Ashland, MA). In parallel, 293 T cells were seeded as a control. Cells were cultured at 37 °C in DMEM, 10% FCS, puromycin 1 µg/ml. The following day, cells were placed into 1 ml incomplete RPMI w/o Phenol red at pH 7.4 with 25 mM HEPES/1% BSA and transferred to the heated (37 °C) stages for confocal microscopy imaging. 7B2-PAC-Alexa488 and sCD4-183 were added to final concentrations of 1 µg/ml each. Taking images of different cells started after the addition of Abs, generally 50 observations during the 90 min period. Pinhole settings were such that an optical slice was less than 1 µm. The intracellular location of Alexa-labeled IT was confirmed by quenching the Alexa488 bound on the surface^45^. After 90 min of imaging, one specific region was analyzed before and after adding 3 mg/ml TB. The excitation was at 488 nm and Alexa488 fluorescence emission was detected by 530 ± 30 band-pass filter. TB emission was detected by 650 ± 13 band-pass filter.

### Confocal microscopy for cell binding and internalization

In one day before confocal experiment, 4 × 10^4^ cell per well of two cell lines, 293 T and Env-transfected-293T cells, were seeded on a multiple-chamber slide (Nalge Nunc International). The next day, in order to block the IT internalization, the media of two control wells were replaced with PBS/BSA/0.01% sodium azide (PBA) 400 µl per well for 45 min in RT. Then, cells were treated by 1 µg Ab/IT in 500 µl PBS or PBA, incubated 60 min in RT. The antibodies were 7B2-Alexa488, 7B2-PAC-Alexa488 and 7B2-RAC-Alexa488 in the presence of 1 µg sCD4-IgG2). The controls were chRAC18 and sCD4-IgG2-Alexa488. One well was left without adding antibody. BFA-bodipy (250 ng/ml) was added to cells to demonstrate rapid accumulation of toxins in Golgi and ER. BFA-bodipy was tested to determine that at the concentration used there was minimal effect of the BFA on the cytotoxicity of ITs^32^. After 2X washing with PBA, the cells were fixed in 2% paraformaldehyde/PBS in 45 min. We took out the solution, and removed the slide box, added one drop SlowFade Gold Mountant DAPI (Life Technologies) and covered it by slide glass. Images were obtained with an inverted Zeiss LSM 510 microscope, a 63 × 1.4 NA oil-immersion objective, and Zeiss LSM software. For each cell, a Z-stack with 1 µm steps was imaged.

### Cytotoxicity measurements for immunoconjugates

A cytotoxicity assay was performed to analyze the cell killing ability of ITs The target H9/NL4-3 cells or Env-transfected 293 T cells (or their uninfected/untransfected parents as controls) were plated in triplicate in Complete RPMI (Supplement RPMI-1640 medium with 10% FCS and 1% sterile antibiotic) or DMEM plus 10% FCS, respectively, in 96 well flat-bottom tissue culture plates (Costar). Controls included: no cells (background) and cells in the absence of IT or in the presence of naked antibody. Serial dilutions of ITs, including the Alexa-labeled and unlabeled versions, were incubated with cells for 3 days. The incubation of 7B2-based ITs (not 924-based ITs) were in the presence or absence of 300 ng/ml of sCD4. For the final 6 hr of incubation, MTS/PMS substrate (Promega, Madison, WI) was added to each well and plates read hourly at 490 nm. Results represent the mean and SEM of triplicate samples (representative of three independent experiments), and are plotted as A_490_ with the no cell background subtracted. The percentage of cell viability was quantified by calculating the ratio below:

1 $$ \% \,\mathrm{of}\,\mathrm{cell}\,\mathrm{viability}=\mathrm{OD490}\,\text{nm}\,(\mathrm{antibody}\,\mathrm{treated}\,\mathrm{cells})/\mathrm{OD490}\text{nm}\,(\mathrm{negative}\,\mathrm{control}\,\mathrm{cells})\times 100 \% $$

### Statistical analyses

Statistical analyses were performed using GraphPad Prism, v5.0. Data are shown as mean and SEM of the indicated number of replicate values. If no error bar appears present, the error bars are smaller than, and obscured by, the symbol. The method for statistical comparison is by unpaired two-tailed Student’s t-test, unless specifically indicated otherwise.

### Data Availability

All data generated or analysed during this study are included in this published article and its Supplementary Information file.

## Electronic supplementary material

Supplementary Information
