# Supplementary material for: Selective cytotoxicity of a novel immunotoxin based on pulchellin A chain for cells expressing HIV envelope
Source: Sci Rep. 2017 Aug 8;7:7579. doi: 10.1038/s41598-017-08037-3 (PMC5548917; doi:10.1038/s41598-017-08037-3)
Supplement: Supplementary file 1 — Supplementary Information [file 41598_2017_8037_MOESM1_ESM.pdf]

Selective cytotoxicity of a novel immunotoxin based on pulchellin A chain for cells expressing HIV envelope

Mohammad Sadraeian[1,2,3], Francisco E. G. Guimarães [1], Ana P. U. Araújo [1], David K. Worthylake[4], Louis LeCour Jr[4], Seth H. Pincus[2,3]

[1] Instituto de Física de São Carlos, Universidade de São Paulo, Caixa Postal 369, São Carlos, SP, CEP 13560-970, Brazil

[2] Research Institute for Children, Children's Hospital, New Orleans, LA 70118, USA

[3] Department of Microbiology, Immunology, and Parasitology, Louisiana State University Health Sciences Center, New Orleans, Louisiana 70112, United States

[4] Department of Biochemistry and Molecular Biology, Louisiana State University Health Sciences Center, New Orleans, Louisiana 70112, United States

* Corresponding authors:

Francisco Eduardo Gontijo Guimarães, email: guimaraes@ifsc.usp.br, +55(16)33739792

Seth Pincus, email: seth.pincus@montana.edu, +1 (303) 815 6967

SUPPLEMENTARY INFORMATION

Production and purification of recombinant toxin A chains.

The recombinant PAC and RAC, containing a 6xHis-tag and tobacco etch virus (TEV) cleavage peptide at the N-terminus, were separately expressed in *E. coli* Rosetta (DE3) as described elsewhere [36]. Recombinant proteins were purified on a 5-ml HisTrap column, eluted by adding increasing concentrations of imidazole, and collected. The 6xHis-tag was removed by TEV protease cleavage, dialyzed, concentrated and purified on a HiPrep 26/60 Sephacryl S-200 column (GE Healthcare). The purified products were analyzed by SDS-PAGE before and after purification by HisTrap Nickel Column, after cleavage by TEV protease, and after purification of His6 tag-removed product by Sephacryl S-200 column (data not shown). Fractions containing purified either PAC or RAC were reduced in 2mM B-mercaptoethanol, pooled, and concentrated to 2 mg/ml and stored at –80°C.

Optimization of LC-SPDP biolinker concentration.

HIV mAb 924 (1 mg) in 0.5 ml PBS, pH 8.0, was mixed separately with 10, 20 and 40-fold molar excess of N-succinimidyl 3-(2-pyridyldithio) propionate with long spacer arm (LC-SPDP) (Pierce Chemical. Rockford. IL). After 2 hr incubation at room temperature, they were passed from Zeba columns equilibrated in PBS.

At the same time, RAC and PAC (1 mg in 0.5 ml) were reduced for 30 min in 50 mM dithiothreitol, then were passed from Zeba columns equilibrated in PBS. The two products, RAC and PAC were mixed separately with 10, 20 or 40-fold molar excess of Antibody-biolinker, concentrated to 0.5 ml and incubated overnight at 4°C. The six individual fractions were

analyzed by Microcapillary Electrophoresis and bicinchoninic acid protein assay to find the optimum conjugation. Figure S1 demonstrates that the conjugates with 40-fold molar excess of biolinker can harbor more A chain toxin.

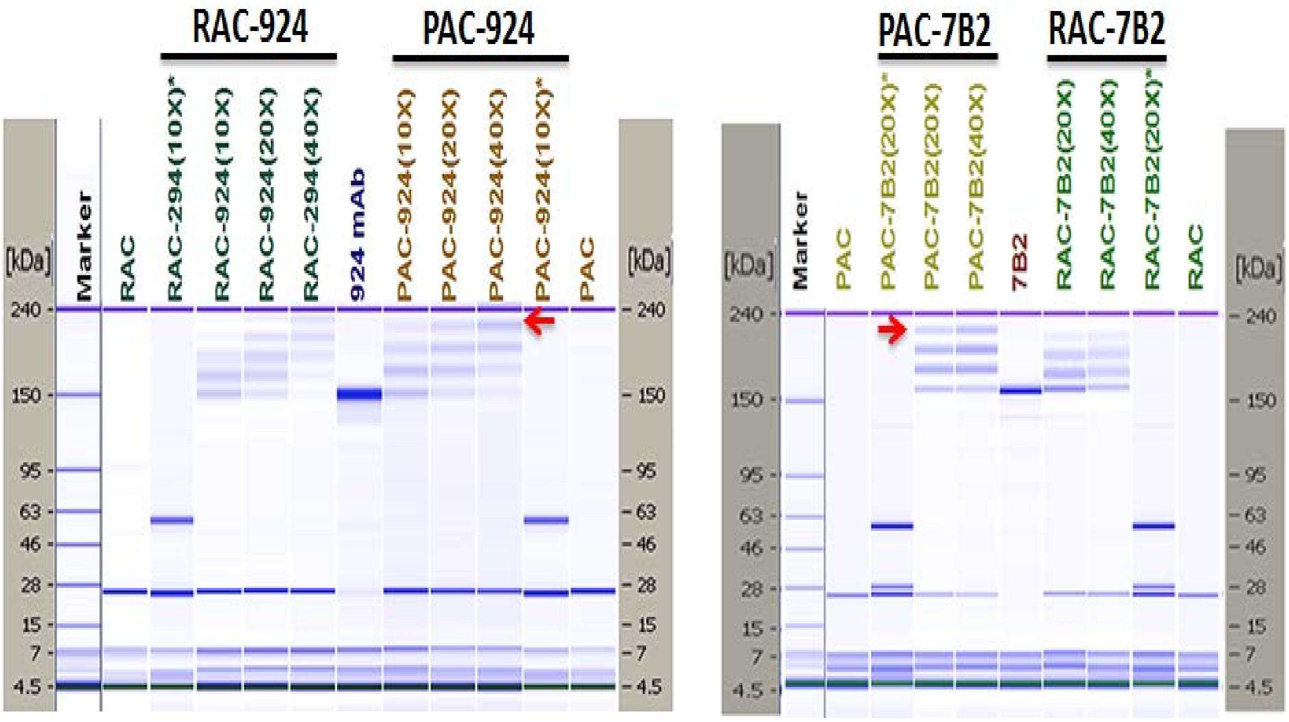

Supplemental Figure S1. Microcapillary electrophoresis of reduced and non-reduced protein preparations with 10, 20 or 40-fold molar excess of antibody-biolinker. Results show the conjugates with 40-fold molar excess of LC-SPDP biolinker give major bands of the predicted molecular weights for one, two and three toxin A chains per antibody molecule. Red arrows indicate the conjugate harboring three toxin A chains. The star symbol (*) means under reducing conditions by using 2-Mercaptoethanol.

The binding ability with different concentration of LC-SPDP (10, 20, 40) were analyzed and compared side by side (Figure S2). The optimization results show that the concentration of biolinker does not have a significant effect on the binding ability of MAbs.

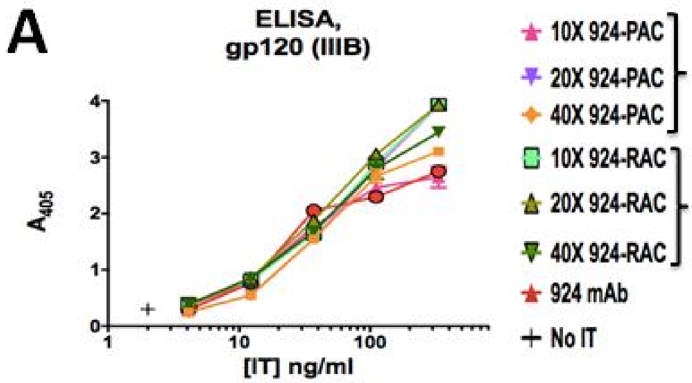

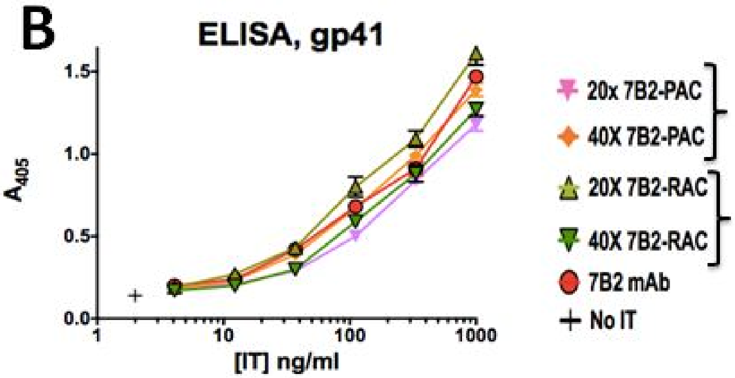

Supplemental Figure S2. The concentration of LC-SPDP biolinker did not have a significant effect on the antigen binding ability of immunoconjugates. The graphs show ELISA assay of the conjugates based on either 924 MAb (**A**) or 7B2 MAb (**B**) with different fold molars of biolinker (10X, 20X or 40X). **A.** ELISA binding of 924-ITs to recombinant gp120 antigen were compared side by side. **B.** 7B2-ITs with 10X or 20X molar excess of biolinker were compared for binding ability to gp41 antigen. Results are representative of at least three independent experiments.
